# Blood Levels of Galectin-9, an Immuno-Regulating Molecule, Reflect the Severity for the Acute and Chronic Infectious Diseases

**DOI:** 10.3390/biom11030430

**Published:** 2021-03-15

**Authors:** Hiroko Iwasaki-Hozumi, Haorile Chagan-Yasutan, Yugo Ashino, Toshio Hattori

**Affiliations:** 1 Department of Health Science and Social Welfare, Kibi International University, Takahashi 716-8508, Japan; hiro_ihz@kiui.ac.jp (H.I.-H.); haorile@gjmyemail.gjmyy.cn (H.C.-Y.); 2 Mongolian Psychosomatic Medicine Department, International Mongolian Medicine Hospital of Inner Mongolia, Hohhot 010065, China; 3 Department of Respiratory Medicine, Sendai City Hospital, Sendai 982-8502, Japan; ashino-yug@hospital.city.sendai.jp

**Keywords:** plasma Gal-9, cleaved Gal-9, disease severity, HIV, dengue, malaria, tuberculosis, AIDS, leptospirosis, COVID-19

## Abstract

Galectin-9 (Gal-9) is a β-galactoside-binding lectin capable of promoting or suppressing the progression of infectious diseases. This protein is susceptible to cleavage of its linker-peptides by several proteases, and the resulting cleaved forms, N-terminal carbohydrate recognition domain (CRD) and C-terminal CRD, bind to various glycans. It has been suggested that full-length (FL)-Gal-9 and the truncated (Tr)-Gal-9s could exert different functions from one another via their different glycan-binding activities. We propose that FL-Gal-9 regulates the pathogenesis of infectious diseases, including human immunodeficiency virus (HIV) infection, HIV co-infected with opportunistic infection (HIV/OI), dengue, malaria, leptospirosis, and tuberculosis (TB). We also suggest that the blood levels of FL-Gal-9 reflect the severity of dengue, malaria, and HIV/OI, and those of Tr-Gal-9 markedly reflect the severity of HIV/OI. Recently, matrix metallopeptidase-9 (MMP-9) was suggested to be an indicator of respiratory failure from coronavirus disease 2019 (COVID-19) as well as useful for differentiating pulmonary from extrapulmonary TB. The protease cleavage of FL-Gal-9 may lead to uncontrolled hyper-immune activation, including a cytokine storm. In summary, Gal-9 has potential to reflect the disease severity for the acute and chronic infectious diseases.

## 1. Introduction

Galectins constitute a protein family of soluble and non-glycosylated animal lectins that show a β-galactoside-binding activity via a conserved sequence of approximately 130–140 amino acids located in the carbohydrate recognition domain (CRD). To date, fifteen members, named in the order of their identification, have been identified in mammals. The galectin family members are classified into three types on the basis of their structures. The prototypical galectins, galectin-1, -2, -5, -7, -10, -11, -13, -14, and -15, each have a single CRD and form non-covalent homodimers. The chimeric type galectin, galectin-3, is composed of an N-terminal non-carbohydrate domain and a C-terminal CRD. The tandem-repeat type galectins, galectin-4, -6, -8, -9, and -12, each consist of two homologous CRDs joined by a linker peptide.

Human galectin-9 (hGal-9) was first identified as a possible antigen in patients with Hodgkin’s disease, suggested to play an important role in the regulation of cellular interactions within the immune system like other galectins [1]. Gal-9 is widely expressed in various tissues and cell types associated with the immune system, and it mediates their functions both intracellularly and extracellularly. This protein has been reported to regulate cell proliferation, differentiation, and apoptosis as well as RNA splicing, cell–cell and cell–matrix adhesion, cellular motility, cell–cell signal transduction, and innate/adaptive immunity [2]. Notably, Gal-9 is a ligand for T cell immunoglobulin and mucin domain-3 (Tim-3), which is expressed on dysfunctional T cells during viral infections. Elevated levels of circulating Gal-9 have been reported in humans infected with various viruses, bacteria, and parasites [3,4,5,6]. In this review, we summarize the role of Gal-9 in the pathogenesis of infectious diseases on the basis of its bioactivity and propose its significance as a new infectious disease severity marker.

## 2. Galectin-9 Biochemistry

### 2.1. Structural Basis and Binding Properties of Gal-9

**Figure 1:**
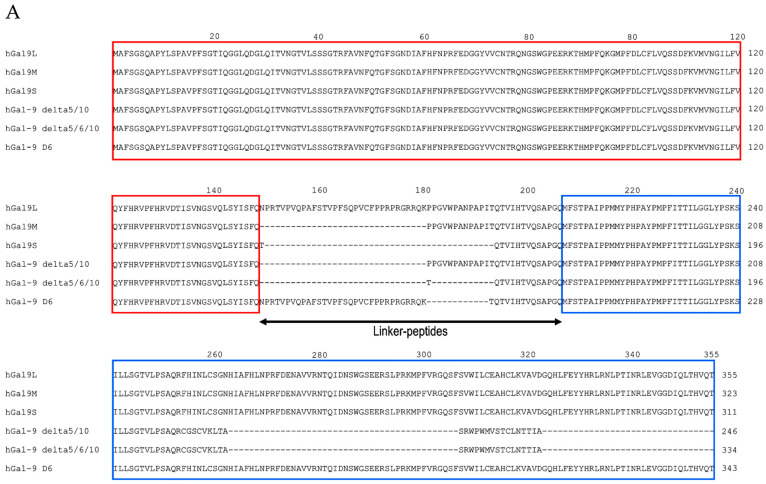

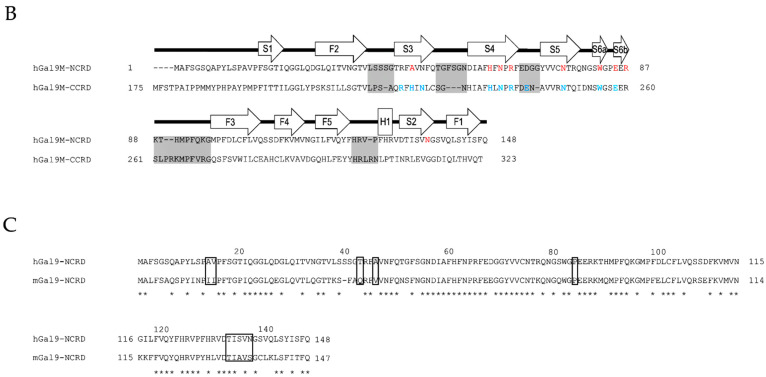
**Amino acid sequences and the secondary structure of hGal-9 (A–C).** Amino acid alignment of hGal-9 isoforms (**A**), the alignment and secondary structures of hGal-9M-NCRD and -CCRD (**B**), and the alignment of hGal-9-NCRD and mGal-9-NCRD (**C**). The all alignment analysis was conducted using Clustal Omega program [http://www.clustal.org/omega/ (Retrieved on 28 December 2020)]. Human Gal-9-NCRD and -CCRD among six isoforms are indicated by red and blue boxes, respectively. The secondary elements are indicated by arrows (β-strand) and a rectangle (α-helix). The loop regions with large deviations between hGal-9-NCRD and -CCRD are highlighted in gray. The carbohydrate-binding residues for the binding of hGal-9-NCRD to lactose, LacNAc trimer (LN3), oligolactosamines, the Forssmann pentasaccharide, and A-hexasaccharide are indicated in red and for the binding of hGal-9-CCRD to BIPA and SiaLac are indicated in blue. Among hGal-9-NCRD and mGal-9-NCRD, the conserved amino acid residues are indicated by asterisk, and the amino acid residues that are responsible for the different target specificities are indicated by black *boxes*.

Wada et al. were the first to report the Gal-9 isoform with 26 amino acid linker-peptides from murine intestines [7]. Subsequently, three isoforms of hGal-9 (hGal-9S, -9M, and -9L) were found in Jurkat cells [8]. Human Gal-9M corresponds to a predominant form with 26 amino acids, and hGal-9S and hGal-9L have 14 and 58 amino acid linker-peptides, respectively (Figure 1A). The transcripts of *LGALS9* gene encoding Gal-9 are subject to alternative splicing. Five splicing variants, hGal-9L, -9M, -9S, delta5/10, and delta5/6/10, are expressed in human endothelial cells, and Gal-9 D6 with these five variants are expressed in decidua and placenta tissues during pregnancy [9,10].

The N-terminal CRD (NCRD) is composed of six-stranded and five-stranded β-sheets and a short helix, which together form a β-sandwich arrangement [11]. The structure formed by the hGal-9 C-terminal CRD (CCRD) is similar to that formed by the NCRD; it consists of a β-sandwich structure formed by two anti-parallel β-sheets with a short α-helix (Figure 1B) [12].

Glycans are involved in a wide range of processes in inflammation and in tumor, microbial, and parasitic pathogenesis. Gal-9 binds to multiple glycosylated molecules and translates glycan-coded information into immune cells. A recent study on the glycan-binding properties of galectin-9 showed that the NCRD and CCRD of Gal-9 have both common and distinct glycan-specificity features and allow Gal-9 to bind to a variety of glycans. The structural analysis demonstrated the crystal structures of the hGal-9-NCRD in a complex with lactose, Forssman pentasaccharide, A-hexasaccharide, and *N*-acetyllactosamine (LacNAc) trimer, and those of the hGal-9-CCRD in its free form or in a complex with LacNAc, the biantennary pyridylaminated oligosaccharide (BIPA), or α-2-3-sialyllactose (SiaLac) [11,12]. The β-galactoside moiety is deeply buried in the site among the NCRD S4-S6 and CCRD S3-S6 β-strand. Another study suggested that recognition of the β-galactoside residue by galectin S4 strands is the most conserved feature of galectin-binding activity [13]. Especially, Trp, His, Asn, Arg, and Glu among these moiety in the both CRDs and Asn137 in S2 β-strand in the NCRD were found to be involved in glycan binding of the NCRD and CCRD (Figure 1B) [11,12]. Apart from S4-S6 β-strand, Ala46 was demonstrated to be responsible for the striking affinity of the NCRD for oligolactosamines, the Forssmann pentasaccharide, and A-hexasaccharide. Structural differences between the NCRD and CCRD were found in their loop regions. Among them, the loop regions of F2-S3, S3-S4, S4-S5, S6b-F3, and F5-H1 deviate greatly. These loops are associated with the different specificities for glycans between the NCRD and CCRD.

A detailed analysis of the glycan-binding specificity of Gal-9 in vitro was performed by frontal affinity chromatography [14]. According to the results, hGal-9 has a high affinity for branched *N*-glycan-type oligosaccharides and β1-3-linked poly-*N*-acetyllactosamines, namely oligolactosamines with a linear structure. Compared with hGal-9-CCRD, the NCRD has a much higher affinity for oligolactosamines and glycolipid-type glycans, Forssman pentasaccharide, and A-hexasaccharide, whereas both CRDs have a similar affinity for branched oligosaccharides. Human Gal-9 has a significant affinity for α-2-3-sialylated oligosaccharides as in the case of other galectins.

In addition to interacting with glycan, Gal-9 can also undergo an intermolecular interaction with other Gal-9 proteins and other galectins [15], and is assumed as forming a series of multimers until it reaches a target receptor. Notably, Gal-9 is thought to be unaffected by carbohydrate modification such as a phosphorylation and cleaving peptide bond because it does not have an N-terminal signal sequence, and consequently, Gal-9 is released via non-classical secretory pathways.

Nagae et al. elucidated that the NCRD of mouse Gal-9 (mGal-9) forms a non-canonical dimer [16], whereas hGal-9-NCRD exists as a monomer, despite its high sequence identity to the mouse homologue [11]. The differing amino acid residues on the concave surface of hGal-9 and mGal-9 were demonstrated to affect their target specificities (Figure 1C), and these differences may explain their differences in cellular function. These results suggest that the functions of Gal-9 described in mice may not be applicable in human cases. To understand the roles of hGal-9 in the pathogenesis of various diseases, further studies using human samples are needed.

### 2.2. Protease-Susceptibility in the Linker-Peptides of Gal-9

Tandem-repeat-type galectins containing Gal-9 are more susceptible to proteolysis compared with other types of galectins owing to the presence of relatively long linker-peptides. The linker peptides of hGal-9M and -9S were remarkably degraded by matrix metallopeptidase (MMP)-3/stromelysin and elastase [17]. They were hardly degraded by trypsin, probably because of the absence of arginine and/or lysine residues. Another study demonstrated that Gal-9L was susceptible to cleavage by thrombin within its linker-peptides, whereas Gal-9M was resistant to such cleavage [18].

**Figure 2:**
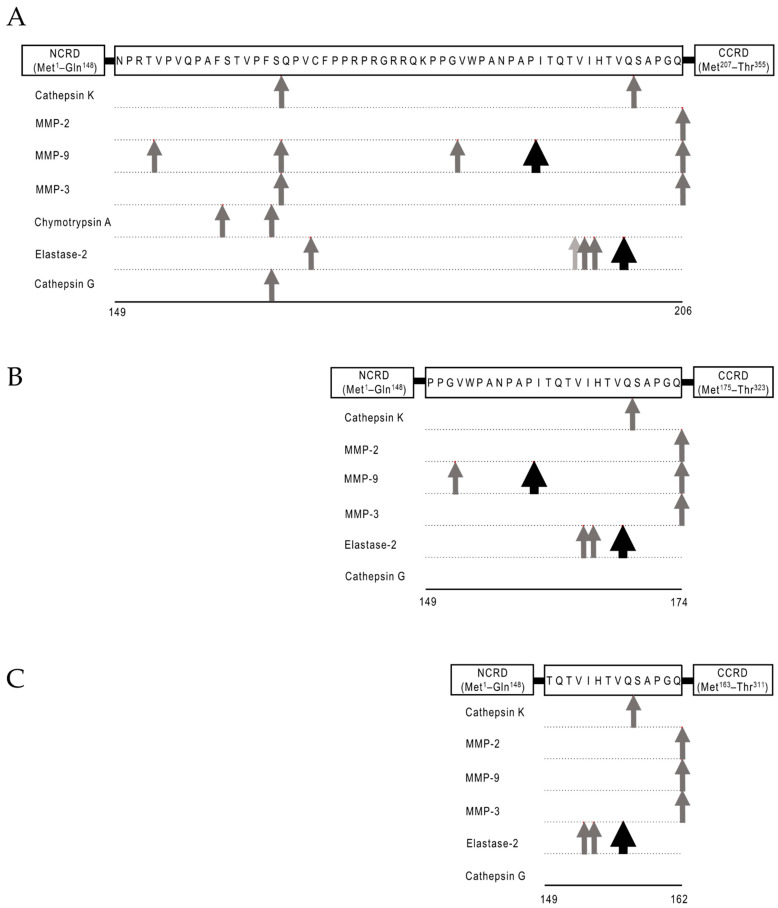
**Predicted hGal-9 cleavage sites (A–C).** Schematic representation of cleavage sites in the linker-peptides of hGal-9L (**A**), hGal-9M (**B**), and hGal-9S (**C**) that were predicted by an analysis conducted using PROSPER. The sequences list the amino acids between the linker-peptides. The arrows indicate the cleavage sites, with their color reflecting the quantitative cleavage probability score for each site as follows, ➡: 1.25 ≤ score <1.5, ➡: 1.0 ≤ score < 1.25, ➡: 0.8 ≤ score < 1.0.

In this review, we demonstrate the predicted cleavage sites by several proteases in the linker-peptides of three Gal-9 isoforms, i.e., the predominant wildtype hGal-9M as well as -9L and -9S, on the basis of an analysis using PROSPER [19], which is a computational tool for the rapid prediction of protease-specific cleavage sites in a substrate (Figure 2). According to the analysis, cathepsin K, MMP-2, MMP-9, MMP-3, chymotripsin A, elastase-2, and cathepsin G are probable candidates for cleaving the linker-peptides of hGal-9. Among these proteases, MMP-9 and elastase-2 were found to have the most cleavage possibility scores for the isoforms. Gal-9M and -9L can be digested by MMP-9 at the P–I bond of the NPAPITQT segment, with a cleavage possibility score of 1.31. All three isoforms can be digested by elastase-2 at the V–Q bond of IHTVQSAP with a cleavage possibility score of 1.37. As expected, it appears that the longer linker-peptides can be cleaved more frequently at more positions by proteases.

Because of the protease susceptibility of Gal-9, it is difficult to analyze the function of this protein in vivo. Nishi et al. developed a protease-resistant Gal-9 (G9Null), which lacks all linker-peptides, that is stable in solution [17]. Gal9Null was able to induce more apoptosis/growth inhibition of tumor cells compared with hGal-9M and -9S at all tested concentrations and has been used for both in vitro and in vivo studies on Gal-9. Furthermore, by modifying the N-terminal region of CCRD, Itoh et al. developed a protease-resistant Gal-9 in which the solubility and yield are both improved compared with G9Null [20]. The biological activity and protease resistance levels of this protease-resistant Gal-9 are better than or comparable with those of Gal9Null.

## 3. Galectin-9 Molecular and Cellular Biology

Gal-9 has been found to have functions in both the innate and adaptive immune systems. Many studies investigating the underlying mechanisms have especially shown that Gal-9 regulates intra- and extra-cellular signal transduction by interacting with multiple receptors that exert the distinct and often opposing biological effects. Here, we discuss Gal-9 interaction with multiple receptors and its regulatory roles affecting cellular functions and immune conditions.

### 3.1. Gal-9 Interaction with Multiple Receptors

#### 3.1.1. Ig Superfamily

Of the identified receptors of Gal-9, Tim-3, a T-cell inhibitory receptor and marker for T-cell exhaustion, has been studied most extensively. Gal-9 binding to Tim-3 induces tyrosine phosphorylation of the cytoplasmic tail of Tim-3 and activates Src family kinases in T cells and monocytes [21,22,23]. Additionally, Gal-9 regulates mast cell function, suppressing its excessive degranulation by binding to the heavily glycosylated immunoglobulin (Ig)E [24].

#### 3.1.2. TNF Receptor Family

Gal-9 binds to several molecules and exhibits a wide variety of functions. One of the molecules bound by Gal-9 is 4-1BB, a cysteine-rich cell surface molecule that interacts with its tumor necrosis factor (TNF) family ligand. The action of agonist anti-4-1BB in suppressing allergic and autoimmune diseases was identified to be dependent on Gal-9 [25]. In that study, Gal-9 directly bound to 4-1BB in a region distinct from the binding sites of the agonist antibodies or natural ligands of 4-1BB. This result suggests that Gal-9 augments 4-1BB aggregation, signaling, and activity in T cells, dendritic cells (DCs), and natural killer cells when 4-1BB is engaged by its agonist or ligand. Additionally, 4-1BB potentiates immune responses against tumors and viruses. The agonist antibodies against 4-1BB were found to enhance anti-tumor activity [26]. 4-1BB promotes immune responses against viruses interacting with its ligand, 4-1BBL [27,28]. Thus, the interaction of Gal-9 with 4-BB may contribute to the suppression of tumors and viruses. Another Gal-9-dependent process is the CD4^+^ forkhead box P3 (Foxp3)^+^ regulatory T cell (Treg) expansion and associated suppression of allergic inflammation by death receptor 3 (DR3), another member of the TNF receptor family [29].

#### 3.1.3. Adhesive Molecule

Furthermore, the cell surface adhesive molecule CD44 can potentially interact with Gal-9; their binding regulates leukocyte migration during allergic lung inflammation [30] and cancer metastasis [31] by inhibiting the CD44–hyaluronic acid (HA) interaction. Notably, Gal-9 expression by induced Treg (iTreg) cells is essential for the generation and function of these cells. Gal-9 increases iTreg stability and function by binding to its receptor, CD44, which forms a complex with activated Smad3 and transforming growth factor-β (TGF-β) receptor 1. Smad3, induced by Gal-9 signaling, activates iTreg cells by upregulating the transcription of *Foxp3* and *Lgals9* [32]. In addition to CD44, vascular cell adhesion molecule-1 (VCAM-1) is another cell surface molecule that interacts with Gal-9; the binding of VCAM-1 to integrin α_4_β_1_ (very late antigen-4, VLA-4) is suppressed by Gal-9. The interaction between VCAM-1 and VLA-4 is critical for the adhesion of tumor cells to endothelium. Thus, Gal-9 suppresses the tumor cell adhesion to extracellular matrix that is necessary for cellular movement in metastasis [31].

#### 3.1.4. Enzyme

Gal-9 also binds to protein disulfide isomerase (PDI), a cell surface enzyme. This binding increases PDI retention on the surface of CD4^+^ Th2 cells and alters the redox status of the plasma membrane; consequently, Gal-9 increases cell migration through the extracellular matrix via β_3_ integrin [33,34].

#### 3.1.5. C-Type Lectin Receptor

Cano et al. identified the function of Gal-9 in actin cytoskeleton reorganization. They also described an interaction between Gal-9 and C-type lectin receptor within both the human and murine DC cytosol [35], which indicates that Gal-9 is an evolutionarily conserved lectin which functions in maintaining the cortical cytoskeleton structure and function of DCs. Furthermore, their study confirmed that intracellular Gal-9 is required for actin polymerization and controls plasma membrane rigidity by enhancing the activity of the actin-binding protein, RAS-related C3 botulinum toxin substrate 1 (Rac-1). These results indicate the intracellular Gal-9 regulates phagocytic activity via a reorganization of the actin cytoskeleton that underlies DC plasma membrane rigidity. Gal-9 may also regulates cell morphology and motility by interacting with the other glycoproteins on the cytoskeleton, organelles, and extracellular matrix.

### 3.2. Immune-Potentiating and -Suppressive Functions of Gal-9

Gal-9 is expressed in endothelial cells, the epithelium of the gastrointestinal tract, and several immune cells, including T cells, B cells, macrophages, mast cells, and DCs. Among them, the regulatory roles of Gal-9 in T-cell development and homeostasis have been mostly elucidated. For example, Gal-9 induces the apoptosis of CD4^+^ T-helper (Th)1 and Th17 cells [36,37,38,39]. On the other hand, Gal-9 activates resting blood T cells in the absence of typical activating signals and expands Th1 cells and central memory T-cells. In the presence of activating signals, Gal-9 does not expand T-cells, but skews the CD4^+^/CD8^+^ balance towards a CD4^+^ phenotype [40]. Exogenous Gal-9 promotes Foxp3^+^ Treg, which are suppressors of excessive immunity [41]. Like exogenous Gal-9, Gal-9^+^ Th1 cells inhibit Th17 development but augment Foxp3^+^ Treg development [42].

Most studies have suggested that Gal-9 exhibits immune-suppressive functions in exaggerated immune conditions [32,36,41]. However, some studies have suggested that Gal-9 exhibits immune-potentiating activities under immune-suppressive conditions [43]. Additionally, endogenous levels of Gal-9 are also elevated in many inflammatory conditions [44,45,46,47,48,49].

## 4. Galectin-9 in a Variety of Infectious Diseases

Because Gal-9 is an immuno-regulating molecule, its circulating concentration could reflect an individual’s immune balance and may be a useful clinical biomarker of various infectious diseases. To date, the plasma or serum levels of Gal-9 have been reported in various infectious diseases according to ELISA systems and multiplex immunoassays, Luminex^®^ and LEGENDplex^TM^ using some types of antibodies to detect Gal-9 (Table 1). Notably, the recent study suggested that the reported Gal-9 concentrations in the blood were widely varied, even within healthy controls, depending on the types of antibodies among different immunoassays [50] For example, with the blood samples, the actual Gal-9 values were different between ELISA produced by R&D Systems (RDS) and GalPharma (GalP). In dengue cases, Luminex^®^ by RDS, in which the same capture and detection antibodies as RDS ELISA are used, indicated Gal-9 levels 26-fold higher compared with those obtained from GalP ELISA in healthy control samples, as shown in Table 1. Additionally, it was demonstrated that GalP ELISA recognizes full-length (FL)-Gal-9 and a linker-less artificial form of Gal-9 [Gal-9(0)], indicating that the ELISA detects only FL-Gal-9. In order to elucidate the function of FL-Gal-9 in vivo, it is more adequate to measure the circulating concentration of Gal-9 using GalP ELISA.

Among the reported blood levels of FL-Gal-9 in patients with infectious diseases, we discuss the regulatory roles of FL-Gal-9 in the pathogenesis of human immunodeficiency virus (HIV) infection, HIV co-infected with opportunistic infection (HIV/OI), dengue, malaria, leptospirosis, and tuberculosis (TB). Moreover, we reveal the correlation between the blood levels of FL-Gal-9 and the disease severity for the acute and chronic infectious diseases according to the data derived from us and other groups. We also explore the roles of the cleaved form of Gal-9 (named truncated Gal-9 [Tr-Gal-9]) in these infectious diseases and the possibility of Gal-9 regulating the pathogenesis of coronavirus disease 2019 (COVID-19) which is now prevalent and causes a great deal of damage to the people in the world. We believe that this review can also contribute to further understanding about the pathogenesis of infectious diseases.

### 4.1. Galectin-9 in Human Immunodeficiency Virus (HIV) Infection

HIV infection eventually induces the immune dysfunction and the pathological syndrome clinically defined as acquired immune deficiency syndrome (AIDS). In 2019, of the estimated 38 million people living with HIV, only 25.4 million people had access to treatment, whereas the other 12.6 million did not [65]. Presently, there are an estimated 690,000 AIDS-related deaths and 1.7 million new infections annually, which are 23% lower than the 2010 estimates.

Antiretroviral therapy (ART) has demonstrated efficacy for suppressing HIV replication in infected patients, and consequently, HIV infection can be managed as a chronic disease. However, ART is unable to achieve complete viral eradication because of the persistence of latently infected long-lived CD4^+^ T cells [66,67]. This persistent infection leads to continuing immune activation, chronic inflammation, and damage to various tissues and organs. HIV-infected patients successfully treated with ART have a higher incidence of cardiovascular, liver, renal, and bone disease, neurocognitive disorders, and cancer as compared with aged-matched HIV-uninfected individuals [68,69]. Interestingly, some HIV-infected elite controllers, which account for less than 1% of untreated HIV-positive patients, are able to suppress HIV without ART, maintaining a viral load of <50 copies/mL [70]. Most elite controllers maintain normal CD4^+^ T cell counts with only a slight decline and a long-term lack of disease progression [71]. However, progressive CD4^+^ T-cell loss has been shown in some elite controllers. Additionally, it has been observed that some elite controllers with declining CD4^+^ T cell levels do progress to clinical AIDS with high levels of T-cell activation levels despite viral control [72,73]. Other studies have also reported that subsets of elite controllers develop overt viremia and CD4^+^ T-cell decline over the course of infection [74]. Therefore, understanding the disease progression in ART-suppressed patients and elite controllers will be essential for developing new strategies for treating patients with HIV infection.

We and other groups have demonstrated that the plasma Gal-9 levels are elevated in HIV-infected patients and that these levels may be involved in HIV pathogenesis (Table 1) [51,52,54,75,76]. In acute HIV infection, a rapid increase of Gal-9 occurring earlier than elevations in other inflammatory markers, such as C-reactive protein (CRP) or serum amyloid acid (SAA), was observed [51]. Gal-9 levels remained significantly elevated in ART-treated HIV-suppressed subjects and elite controllers compared with age-matched HIV-negative control subjects during chronic infection [52]. That study also found an elevated frequency of Tim-3-expressing CD8^+^ T cells in both ART-suppressed patients and elite controllers. Other studies have demonstrated that Tim-3^+^CD8^+^ T cells, the levels of which were increased in progressive HIV infection, lead to CD8^+^ T cell dysfunction through Gal-9 interaction [77,78]. Therefore, Tandon et al. proposed that high levels of Gal-9 contributing to CD8^+^ T cell dysfunction via Tim-3 may lead to T cell-driven immune exhaustion and contribute to persistent inflammation during viral suppression in ART-suppressed patients and elite controllers [52].

More recent studies have reported that the surface expression of Gal-9 on natural killer (NK) cells and T cells affects chronic immune activation in HIV infection. One study demonstrated that up-regulation of Gal-9 on NK cells in HIV-infected groups compared with healthy control subjects and expansion of T-cell immunoreceptor with immunoglobulin and ITIM domains (TIGIT)^+^ NK cells in HIV-infected individuals [79]. Additionally, they found that Gal-9^+^ NK cells exhibited the impaired expression of cytotoxic mediators and enhanced IFN-γ production, whereas TIGIT^+^ NK cells express higher amounts of cytotoxic mediators and lower IFN-γ in HIV-infected individuals. These findings suggested that over expression of the surface Gal-9 on NK cells impairs their cytotoxicity but enables them to secret more IFN-γ, which may contribute to increased immune activation seen in chronic HIV infection although this is different from the previous results of recombinant Gal-9 to impair the functions which contain cytotoxicity and IFN-γ production, of NK cells [80]. Meanwhile, another study reported significantly higher expression of surface Gal-9 and V-domain Ig suppressor of T cell activation (VISTA) on both CD4^+^ and CD8^+^ T cells in HIV infected patients compared with healthy control subjects. Those expression was associated with impaired T cell effector functions. Gal-9 was co-expressed with other coinhibitory receptors such as TIGIT, CD160, CD39, VISTA, and PD-1. Among them, co-expression of Gal-9 with PD-1 was associated with a more terminally exhausted T cell phenotype in HIV infected patients [81].

The increase of HIV RNA in plasma is associated with a depletion of memory CD4^+^ T cells [82]. It has been suggested that a cytokine storm, with elevated levels of interferon (IFN)-α, IFN-γ, interleukin (IL)-15, IFN-γ-inducible protein-10 (IP-10), TNF-α, monocyte chemotactic protein-1 (MCP-1), IL-6, and IL-18, is occurring during acute HIV infection [83] and may contribute to CD4^+^ T cell apoptosis [84]. Gal-9 was identified as a critical molecule in the first wave of the cytokine storm [52]. Moreover, indirect CD4^+^ T cell killing can result in CD4^+^ T cell depletion during HIV infection [85,86]. Recent studies have highlighted the opposing roles of Gal-9 in HIV infection. One study showed that Gal-9 induced HIV entry into CD4^+^ T cells via PDI in a Tim-3-independent manner, resulting in increased viral replication [33]. Whereas, another study found that Tim-3^+^CD4^+^ T cells by ligation of Gal-9 makes CD4^+^ T cells less susceptible to HIV by downregulating CCR5 and CXCR4, and additionally, p21 upregulation by Gal-9 reduces infection of CD4^+^ T cells to HIV-1 via Tim-3 [87].

Gal-9 is known to induce T cell receptor (TCR) signaling [88]. One recent study showed that treatment of CD4^+^ T cells with recombinant Gal-9 reverses HIV latency both in vitro and ex vivo and induces HIV transcription [89]. That study also found that the levels of endogenous Gal-9 in plasma of ART-suppressed patients are associated with HIV transcription. Colomb et al. reported that Gal-9 modulates HIV transcription through activating the TCR-downstream extracellular signal-regulated kinase (ERK) and cAMP response element binding protein (CREB) pathways in a lymphocyte-specific protein tyrosine kinase (Lck)-dependent manner and that those pathways induce a proinflammatory response, i.e., the secretion of IL-2 and TNF-α, consequently, activating CD4^+^ T cells [90]. Their study also demonstrated that the proinflammatory response could be inhibited by treatment with rapamycin, an inhibitor of mammalian target of rapamycin (mTOR), without impacting Gal-9-mediated viral reactivation. These findings may have implications for understanding how endogenous Gal-9 influences the maintenance of chronic immune activation during ART-suppressed HIV infection. Very recently, it was proposed that plasma Gal-9 levels can be used as a surrogate marker of viremia in HIV-infected patients on ART, which has cost implications for HIV management and could be particularly helpful in resource-limited settings [91].

In 2016, the median TB incidence rate for HIV-infected patients was 22-fold higher than that for uninfected people living in the same country [92]. In HIV-infected individuals, chronic immune activation, associated with T cell exhaustion which interacts with “inflammaging”, could be a cause of its vulnerability to TB [93,94,95,96]. Consequently, they develop extrapulmonary TB with a paucity of bacteria in their sputa. As mentioned above, Gal-9 could contribute to T cell exhaustion in chronic HIV patients. Recently, an assay to measure the cleaved form of Gal-9 was developed [50]. A biomarker analysis conducted in patients with HIV/OI revealed that levels of Tr-Gal-9 markedly reflect the disease severity of the co-infected individuals [53,97]. The Tr-Gal-9 may be generated via cleavage by MMP-9, which has also been proposed to be a non-sputum-based biomarker for differentiating pulmonary TB and extrapulmonary TB [98].

### 4.2. Galectin-9 in Dengue Virus Infection

Dengue is a viral infection transmitted from human to human by *Aedes* mosquitos. There were an estimated 96 million apparent and 294 million inapparent cases of dengue virus (DENV) infection globally in 2010 [99]. DENV infections are categorized into three groups: undifferentiated fever, dengue fever (DF), and dengue hemorrhagic fever (DHF) [100]. DHF is further classified into four severity grades, the most severe of which is dengue shock syndrome (DSS) [101]. The course of dengue illness proceeds through the following three phases: febrile, critical, and recovery. Patients infected with DENV typically develop a sudden high-grade fever with increased viremia. This acute febrile phase usually lasts 2–7 days after an incubation period of 4–10 days. During the critical phase, the body temperature drops and typically remains low through days 3–7 of illness, with an increase in capillary permeability in parallel with increasing hematocrit levels.

Ho et al. showed that DENV could infect DCs and that DCs secrete TNF-α and IFN-α, which probably lead to cell maturation after infection [102]. It was reported that endothelial cells infected with DENV in vitro induce cytokine production and nuclear factor kappa B (NF-κB) activation, which may lead to cell death via complement activation [103]. Warke et al. identified the enhanced expression of Gal-9 at 24 h post infection in DENV-infected human umbilical vein endothelial cells, which might be induced by the IFN-α/β pathway and also activated by the IL-1β pathway [104]. Both proinflammatory and anti-inflammatory cytokines and chemokines are induced during the progression of DENV infection, suggesting that multifunctional mediators are involved in the associated pathogenesis [105,106,107,108]. Our group was the first to demonstrate the dynamic release of Gal-9 in acute DENV infection [3]; plasma levels of Gal-9 were significantly elevated in patients during the critical phase of acute DENV infection compared with those in patients suffering from a non-dengue febrile illness or healthy control subjects (Table 1), and these levels were significantly decreased during the recovery phase. The increase of Gal-9 levels in patients with dengue appears to be associated with disease severity (Table 2). Moreover, we showed that DENV content can regulate the increase of circulating Gal-9, which is inversely correlated with monocyte percentages, via a diverse cytokine and chemokine storm. Other work demonstrated that DENV induced Gal-9 secretion as a danger response; Gal-9 and other inflammatory factors, along with their stimulated effector responses, may have subsequently limited further viral replication [109]. Another study showed that Gal-9 and galectin-3BP might be critical inflammatory mediators in acute DENV infection [55]. Recent study showed that up-regulation of Gal-9 in DENV-infected human DCs induces DC migration via interactions with non-Tim-3 receptors [110]. The mechanisms seem to involve the up-regulation of IL-12 and CCR7-mediated activation of mitogen-activated protein kinases (MAPKs), especially ERK.

### 4.3. Galectin-9 in Malaria

Malaria is one of the most common life-threatening diseases, especially in epidemic tropical and subtropical regions. It is caused by *Plasmodium* parasites that are transmitted to humans through infected female *Anopheles* mosquitoes. There are five species of *Plasmodium* that cause malaria in humans, of which *Plasmodium falciparum* and *Plasmodium vivax* pose the greatest threat. There were an estimated 228 million cases of malaria and 405,000 malaria-related deaths globally in 2018 [111].

The circulating levels of TNF-α, IL-6, IL-10, and granulocyte-colony stimulating factor (G-CSF) were found to be elevated in patients with malaria [112,113]. Recent studies have suggested that CD8^+^ T cells may play an important role during the blood-stage of infection by eliminating parasites [114,115]. *P. vivax* infection reduces the total number of CD8^+^ T cells, especially memory cells, during the blood-stage of infection but enhances the number of memory CD8^+^ T cells expressing IL-10, TNF-α, and IFN-γ [114]. Additionally, it was indicated that the loss of effector CD8^+^ T cells mediated by PD-1 during acute malaria can contribute to a loss of long-lived protective memory CD8^+^ T cells that are capable of rapid expansion in response to new infection [115]. Meanwhile, the expression levels of Tim-3 and Gal-9 were found to be upregulated in both the liver and lung of mice following damage induced by infection with *Plasmodium borghei* [116,117]. Our group was the first to reveal that plasma levels of Gal-9 during malaria infection were significantly elevated at day 0 of illness, as compared with days 7 and 28 of illness (Table 1), and that these levels were correlated with the plasma levels of inflammatory cytokines (IFN-α, IFN-γ, TNF, and IL-6) and chemokines (MIP-1β, MCP-1, and Fractalkine). Median Gal-9 levels were also significantly higher in severe malaria (SM) cases compared with uncomplicated malaria (UM) cases and in patients with a blood urea nitrogen to creatinine ratio (BUN/creatinine) of ≥20 mg/dL compared with patients with a BUN/creatinine of <20 mg/dL (Table 2). These findings indicate that Gal-9 levels reflect the severity of malaria [5].

Disruption of the blood–brain barrier (BBB) is responsible for the development of cerebral malaria. Recently, the interaction between CD146 and Gal-9 was reported to contribute to the aggregation of infected red blood cells and lymphocytes in a mouse experimental cerebral malaria system, indicating that the CD146–Gal-9 interaction could be a novel target for treating cerebral malaria [118].

### 4.4. Galectin-9 in Leptospirosis

Leptospirosis is a neglected zoonotic disease with a global distribution. About 5%–10% of patients with leptospirosis can potentially develop severe forms of the disease, of which Weil’s disease has a fatality rate of >10% and leptospirosis pulmonary hemorrhage syndrome has a fatality rate as high as 70%. The most severe form of the disease, Weil’s disease, manifests as severe lung injuries (diffuse alveolar hemorrhage, pulmonary edema, acute respiratory distress syndrome, or a combination of these features) which are accompanied by acute kidney injury (AKI) [119,120]. Lipopolysaccharide found on the outer membranes of Gram-negative bacteria, enhances expression of Gal-9 mRNA and protein during bacterial infection [121]. The leptospiral outer membrane constituents (lipoprotein 32 and *Leptospira* surface adhesin) were shown to activate macrophages through the toll-like receptor (TLR) pathway and establish the predominant signaling component for macrophages through this pathway [122].

In our previous study, a receiver operating characteristic (ROC) curve analysis revealed that plasma levels of Gal-9 had the greatest ability to discriminate patients with leptospirosis from healthy control subjects as compared with other markers (AUC: 0.899) (Table 1) [64]. This finding may indicate that the plasma levels of Gal-9 could reflect the severity of leptospirosis, as they have been reported to do for dengue, malaria, and HIV/OI, given that cytokinemia is also frequently seen in this disease. The recent studies proposed that Gal-9 act as immune checkpoint inhibitors [123,124]. A degradation of Gal-9 by proteases may inactivate the inhibitory activities and lead to uncontrolled hyper-immune activation such as a cytokine storm.

### 4.5. Galectin-9 in Tuberculosis (TB)

TB is caused by infection with *Mycobacterium tuberculosis*, which is spread from human to human through the air via the coughing, sneezing, and spitting of individuals with pulmonary TB. An estimated 10 million people globally fell ill with TB in 2019 [125]. That year, there were an estimated 1.2 million TB-related deaths among HIV-negative patients along with 208,000 deaths among HIV-positive patients. Those infected with HIV are 18 times likely to develop active TB disease compared with people not infected with HIV. Drug-resistant TB is also a public health problem. Around 500,000 people globally developed rifampicin-resistant TB (RR-TB), of which 78% was multidrug-resistant TB (MDR-TB), in 2019.

In mice, Tim-3 interaction with Gal-9, which is expressed by *M. tuberculosis*-infected macrophages, promoted macrophage activation and caspase-1-dependent IL-1β secretion and inhibited intracellular bacterial growth [126]. TNF secretion and TNF receptor 1 cell surface expression in macrophages are directly upregulated by IL-1β, and they induce caspase-3 activation, which restricts the intracellular growth of *M. tuberculosis* [127].

CD4^+^ helper T cells and cytotoxic CD8^+^ T cells (CTLs) become functionally exhausted during *M. tuberculosis* infection. T cell exhaustion, which was first identified in a lymphocytic choriomeningitis virus (LCMV)-infected mice suffering from specific CD8+ T cell dysfunction [128,129], was later confirmed in hepatitis B virus (HBV), hepatitis C virus (HCV), HIV, and cancer patients. The functions of cytotoxicity and effector CD8^+^ T cells lose effector functions such as production of cytokines, capacity to proliferate, cytotoxicity for killing pathogen infected cells, and the generation of effective memory cells in a response to sustained pathogen exposure in chronic infections. CTL exhaustion has been primarily accompanied with upregulation of multiple co-inhibitory receptors such as PD-1, CTLA-4, Lag-3, Tim-3, TIGIT, VISTA, BTLA, 2B4, and CD160 [130]. During chronic M. tuberculosis infection, Tim-3 expressing CD4^+^ and CD8^+^ T cells co-express other inhibitory receptors including PD-1, Lag-3, and 2B4, produce less pro-inflammatory cytokines including IFN-γ, TNF, and IL-2 but more inhibitory cytokine, IL-10, and result in being functionally exhausted [131]. Furthermore, it was demonstrated that the exposure to lipoarabinomannan (LAM), a lipid virulence factor secreted by *M. tuberculosis*, of monocytes during differentiation toward macrophages downregulates their Gal-9 expression, consequently leading to the development of monocyte-derived macrophages (MDM) with a reduced capacity to respond to inflammatory stimuli and restrict *M. tuberculosis* growth [132]. Recent study indicated that the plasma levels of Gal-9 in TB patients was significantly higher compared with healthy control subjects (Table 1) and proposed Gal-9 as a biomarker for TB [53].

### 4.6. Galectin-9 in Coronavirus Disease 2019 (COVID-19)

Accumulating evidence suggests that a subgroup of patients with severe COVID-19 develop cytokine release syndrome (CRS; also known as cytokine storm syndrome), which is characterized by high levels of circulating proinflammatory cytokines that result in direct tissue injury, especially in the lungs [133]. Such patients display significantly elevated levels of proinflammatory or anti-inflammatory cytokines, including Th1 and Th2 cytokines, chemokines, and galectins, including the expected high levels of Gal-9 [62]. Synthesized FL-Gal-9, acting as an immune check molecule, may suppress the synthesis of proinflammatory molecules. MMP-9 has been reported as a potential early indicator of respiratory failure in patients with COVID-19 [134]. Notably, MMP-9 can cleave FL-Gal-9, and this cleavage may cause CRS. (Figure 2). Whereas, Gal-9 was suggested to be associated with the impaired T cell function contributing to reduced antiviral T cell immunity in COVID-19 [63]. We have successfully treated COVID-19 pneumonia with the anti-IL-6 receptor antibody tocilizumab [135]. Investigations on the effects of various drugs on the levels of FL-Gal-9 and Tr-Gal-9 in patients with COVID-19 are underway.

Thrombotic phenomena or diffuse damage is frequently seen in the patients with COVID-19. Both patients with mild and severe cases of COVID-19 had significantly fewer apoptotic CD146^+^ circulating endothelial cells as compared with healthy control subjects [136]. The interaction between these cells and Gal-9 may cause coagulopathy involving red blood cells, as observed for cerebral malaria.

## 5. Conclusions

We demonstrated that the Gal-9 levels in the peripheral blood are elevated in the patients with the acute and chronic infectious diseases and they reflect the disease severity of dengue, malaria, and HIV/OI. The elevation of blood Gal-9 in patients indicates the two possibilities that Gal-9 is one of the causative factor of the pathogenesis and potentiates the disease progression, or that Gal-9 suppresses the development of the disease. Many studies have suggested that Gal-9 plays the opposing roles to promote or inhibit immune activation and inflammation in immune system. In addition to the roles of Gal-9 in the acute diseases, according to the studies on the disease progression in HIV and TB, Gal-9 could be strongly associated with a chronic immune activation induced by T cell exhaustion and other immune dysregulation. Because the upregulation of Gal-9 with multiple co-inhibitory receptors induced by sustained antigen exposure and TCR signaling has been suggested to contribute to a chronic immune activation, the clarification of the roles of Gal-9 in T cell exhaustion and immune dysregulation could be a key for finding out the pathophysiology of the infectious diseases. To find out the most important pathological roles of Gal-9, a closer look at the correlation with clinical findings and studies of Gal-9 secreting cells in each disease would be necessary. It is also important to know if the elevated levels of Gal-9 are involved in immunosuppression and/or coagulopathy in COVID-19 infection.

The plasma levels of Tr-Gal-9 as well as the FL-Gal-9 have been reported to markedly reflect the severity of HIV/OI. The pathophysiology of HIV/OI, of which symptoms worsen rapidly, is considered to be associated with the acute immune activation. Meanwhile, the chronic immune activation and dysregulation of T cell homeostasis are also suggested to be associated with its progression, according to the previous report on HIV/TB. Therefore, FL- and Tr-Gal-9 may exert the function in the acute and chronic disease progression of HIV/OI. In the present review, several candidates of the possible cleavage sites were demonstrated. We need to clarify precisely the functions of Tr-Gal-9 in the pathophysiology of infectious diseases.

In summary, we propose that the blood Gal-9 levels have potential to reflect the disease severity in infectious diseases. Further clarification of the roles of Gal-9 in the pathogenesis of acute and chronic infectious diseases is an essential key for developing clinical potential.

## Figures and Tables

**Table 1:** Comparison of Gal-9 levels in peripheral blood among patients with various infectious diseases.

Disease.	Gal-9 [pg/mL]		*p* Value	Stat.	Statistical Analysis	Sample Type	Immuno Assay	Description	Ref.
	Patients	Control							
HIV infection	Acute HIV **4196** (n = 3)	HC <**46** (n = 30)	Data not shown	Median	Data not shown	Plasma	ELISA (GalP)		[51]
	Chronic HIV **325.6** (n = 58)	HC **54** (n = 19)	<0.0001	Median	Mann-Whiney *U* test	Plasma	ELISA (GalP)		[52]
	HIV/TB **567** (n = 33)	HC **55.5** (n = 30)	<0.0001	Median	Mann-Whiney *U* test	Plasma	ELISA (GalP)		[53]
	HIV/OI* 650 (n = 24)		<0.0001						
	Early untreated HIV **1000** (n = 11)	HC **160** (n = 13)	NS	Median	Mann-Whiney *t* test	Plasma	ELISA (Uscn)	Values were read from the figure	[54]
Dengue	**1525** (n = 65)	HC **196** (n = 30)	<0.001	Mean	Mann-Whiney test	Plasma	ELISA (GalP)		[3]
	**10,287** (n = 187)	HC **5061** (n = 20)	<0.0001	Median	Mann-Whiney *U* test	Serum	Multiplex (RDS)		[55]
HCV infection	Chronic HCV **1276** (n = 22)	HC **112** (n = 10)	0.0005	Mean	Two tailed Mann-Whiney test	Plasma	ELISA (GalP)	Patients: SD = 1503 HC: SD = 210	[56]
	Chronic HCV **146** (n = 50)	HC **0** (n = 39)	0.05	Median	Mann-Whiney test	Serum	ELISA (GalP)		[57]
HBV infection	CHB (ALT>100 IU/L) **14,000** (n = 9)	HC **5700** (n = 10)	0.02	Mean	Mann-Whiney test	Serum	ELISA (Uscn)	Values were read from the figure	[58]
Influenza infection	**184** (n = 43)	HC **14** (n = 20)	<0.05	Mean	Two tailed Mann-Whiney U test	Plasma	ELISA (GalP)	Values were read from the figure	[59]
HCMV infection	HCMV+ reactivators **24,400** (n = 3)	HCMV- **8800** (n = 3)	<0.001	Mean	Bonferroni Dunn test	Plasma	ELISA (RDS)	Day-53 post transplant	[60]
CHIKF	**2192** (n = 44)	HC **46.88** (n = 49)	<0.0001	Median	Mann-Whiney *U* test	Serum	ELISA (RDS)		[61]
COVID-19	**24,770** (n = 23)	HC **6902** (n = 15)	<0.0001	Mean	Two tailed Mann-Whiney test	Plasma	Multiplex (RDS)	Patients: SD = 7512.62 HC: SD = 1551.97	[62]
	Active COVID-19 **2,250,000** (n = 65-92)	Recovered COVID-19 **500,000** (n = 47-66)	<0.0001	Mean	Krustal-Wallis test and Dunn’s test	Plasma	Multiplex (BioLegend)	Values were read from the figure	[63]
		HC **450,000** (n = 24-43)	<0.0001						
Malaria	Day-0 illness **686.5** (n = 50)	Day-28 illness **243** (n = 50)	<0.0001	Median	Mann-Whiney test	Plasma	ELISA (GalP)		[5]
Leptospirosis	**613** (n = 111)	HC **196** (n = 30)	<0.0001	Median	Mann-Whiney *U* test	Plasma	ELISA (GalP)		[64]
TB	**358** (n = 49)	HC **55.5** (n = 30)	<0.0001	Median	Mann-Whiney *U* test	Plasma	ELISA (GalP)		[53]
	**171,500** (n = 36)	HC **14,000** (n = 19)	0.0002	Median	Mann-Whiney *U* test	Plasma	ELISA (RDS)	Patients: active PTB	[4]

The Gal-9 concentrations in the plasma or serum from patients and healthy control (HC) are shown as the median or mean values. HIV: human immunodeficiency virus, TB: tuberculosis, HIV/TB: HIV co-infected with TB, HCV: hepatitis C virus, HBV: hepatitis B virus, CHB: chronic HBV infection, ALT: alanine aminotransferase, HCMV: human cytomegalovirus, CHIKF: Chikungunya fever, COVID-19: coronavirus disease 2019, PTB: pulmonary TB, HC: healthy control, GalP: GalPharma, RDS: R&D Systems, Multiplex (RDS): Liminex^®^, Multiplex (BioLegend): LEGENDPlex^TM^, SD: standard deviation, and NS: not significant. * HIV co-infected with opportunistic infection (OI) other than TB.

**Table 2:** The Gal-9 levels in peripheral blood reflect the severity of infectious diseases.

Disease	Gal-9 [pg/mL]		*p* Value	Stat.	Statistical Analysis	Sample Type	Immuno Assay	Description	Ref.
HIV infection	Non-contollers **555.6** (n = 20)	Elite-controllers **250** (n = 20)	NS	Median	Mann-Whiney *U* test	Plasma	ELISA (GalP)	Values of Elite-cont. and ART-suppressed were read from the figure	[52]
		ART-suppressed **200** (n = 20)	NS						
	Aviremic **4000** (n = 62)	Viremic **11,000** (n = 43)	<0.0001	Data not shown	One tailed Mann-Whiney test	Plasma	ELISA (RDS)	Values were read from the figure	[91]
	Untreated chronic HIV **398** (n = 47)	Early untreated HIV **1000** (n = 11)	<0.001	Median	Mann-Whiney *t* test	Plasma	ELISA (Uscn)	Values were read from the figure	[54]
	HAART-treated HIV **398** (n = 15)		0.0009						
HIV/OI *	Alive **554** (n = 21)	Dead **1042** (n = 3)	0.0301	Median	Mann-Whiney *U* test	Plasma	ELISA (GalP)		[53]
HIV/TB	HIV/LTBI **3500** (n = 17)	HIV/PTB **11,290** (n = 14)	≤0.001	Median	Krustal-Wallis test	Plasma	ELISA (RDS)		[97]
Dengue	DF **1407** (n = 53)	DHF **2464** (n = 12)	Data not shown	Mean	Mann-Whiney test	Plasma	ELISA (GalP)		[3]
HCV infection	HCV infection alone **715** (n = 15)	HCV infection with hepatocellular carcinoma **1376** (n = 7)	Data not shown	Median	Two tailed Mann-Whiney test	Plasma	ELISA (GalP)		[56]
	SVR after treatment **20.8** (n = 24)	chronic HCV infection **146** (n = 50)	0.05	Median	Mann-Whiney test	Serum	ELISA (GalP)		[57]
HBV infection	CHB (ALT<50 IU/L) **6000** (n = 16)	CHB (ALT>100 IU/L) **14,000** (n = 9)	0.01	Mean	Mann-Whiney test	Serum	ELISA (Uscn)	Values were read from the figure	[58]
HCMV infection	HCMV+ non- reactivators **14,100** (n = 3)	HCMV+ reactivators **24,400** (n = 3)	<0.01	Mean	Bonferroni Dunn test	Plasma	ELISA (RDS)	Day-53 post transplant	[60]
Malaria	UM (n = 41) **617** **348**	SM (n = 9) **923** **659**	0.03 0.02	Median	Mann-Whiney test	Plasma	ELISA (GalP)	Day-0 illness Day-7 illness	[5]
	BUN/creatinine <20 (mg/dL) **576.2** (n = 28)	BUN/creatinine ≥20 (mg/dL) **817.3** (n = 22)	0.007					Day-0 illness	
TB	LTBI **1190** (n = 22)	EPTB **6800** (n = 33)	≤0.001	Median	Krustal-Wallis test with Dunn's test	Plasma	ELISA (RDS)		[97]
		PTB **5900** (n = 21)	≤0.001						

The Gal-9 concentrations in plasma or serum are shown as median or mean values. ART: antiretroviral therapy, HARRT: highly active antiretroviral therapy, LTBI: latent TB infection, EPTB: extrapulmonary TB, DF: dengue fever, DHF: dengue hemorrhagic fever, SVR: sustained virologic response, UM: uncomplicated malaria, SM: severe malaria, and BUN: blood urea nitrogen. * HIV co-infected with OI other than TB.
